# Correlation analysis between jejunum metabolites and immune function in Saba and Landrace piglets

**DOI:** 10.3389/fvets.2023.1069809

**Published:** 2023-03-16

**Authors:** Yang He, Yongxiang Li, Yangsu Pan, Anjian Li, Ying Huang, Qianhui Mi, Sumei Zhao, Chunyong Zhang, Jinming Ran, Hong Hu, Hongbin Pan

**Affiliations:** ^1^Yunnan Provincial Key Laboratory of Animal Nutrition and Feed Science, Faculty of Animal Science and Technology, Yunnan Agricultural University, Kunming, China; ^2^College of Modern Agriculture, Dazhou Vocational and Technical College, Dazhou, China

**Keywords:** Saba piglets, Landrace piglets, jejunum chyme, immunity, metabolomics

## Abstract

The immune function of the intestinal mucosa plays a crucial role in the intestinal health of hosts. As signaling molecules and precursors of metabolic reactions, intestinal chyme metabolites are instrumental in maintaining host immune homeostasis. Saba (SB) pigs, a unique local pig species in central Yunnan Province, China. However, research on jejunal metabolites in this species is limited. Here, we used immunohistochemistry and untargeted metabolomics by liquid chromatography mass spectrometry (LC-MS/MS) to study differences in jejunal immunophenotypes and metabolites between six Landrace (LA) and six SB piglets (35 days old). The results showed that the levels of the anti-inflammatory factor interleukin 10 (IL-10) were markedly higher in SB piglets than in LA piglets (*P* < 0.01), while the levels of the proinflammatory factors IL-6, IL-1β, and Toll-like receptor 2 (TLR-2) were markedly lower (*P* < 0.01). Furthermore, the levels of mucin 2 (MUC2) and zona occludens (ZO-1), which are related to mucosal barrier function, were significantly higher in SB piglets than in LA piglets (*P* < 0.01), as were villus height, villus height/crypt depth ratio, and goblet cell number (*P* < 0.05). Differences in jejunal chyme metabolic patterns were observed between the two piglets. In the negative ion mode, cholic acid metabolites ranked in the top 20 and represented 25% of the total. Taurodeoxycholic acid (TDCA) content was significantly higher in SB piglets than in LA piglets (*P* < 0.01). TDCA positively correlated with ZO-1, villus height, villus height/crypt depth ratio, and goblet cell number. These results suggest that SB pigs have a strong jejunal immune function and that TDCA was positively regulates jejunal immunity and mucosal barrier function. Our findings provide a reference for understanding intestinal immune function in different pig breeds and for the discovery of potential biomarkers to help solve health issues related to pig production.

## 1. Introduction

The gut is critical for the digestion and absorption of nutrients, is the largest immune organ in the body, and is in a state of continuous and controllable inflammation (1–3). Intestinal injury and epithelial barrier dysfunction can increase cell bypass permeability, potentially allowing toxins, allergenic compounds, and bacteria to enter the bloodstream, leading to inflammation, immune responses, and systemic issues (4–6). The proximal end of the small intestine is a central site for the digestion and absorption of a large amount of nutrients, and intestinal health plays an important role in these processes in animals (7). The jejunum structurally and functionally connects the upper (duodenum) and lower (ileum) small intestines. The studies have shown that the jejunum can evaluate intestinal health and plays a role in controlling systemic metabolic responses (8, 9).

Pigs are not only a primary source of meat for humans but also an ideal animal model for studying human diseases. In pig production, the sow slaughter cycle is shortened, and reproductive performance is improved through early weaning strategies (10). However, various stressors encountered by piglets during weaning can lead to transient anorexia, intestinal inflammation, intestinal dysbiosis, and diarrhea, which remains a prominent problem in the pig industry (11, 12). Landrace (LA) pigs are a well-known and widely distributed pig breed, noted for their fast growth and high feed utilization, but also for their high feeding and management requirements, weak physique, poor stress resistance, and reproductive difficulties (6). In contrast, Saba (SB) pigs, an excellent native pig breed from Yunnan Province, China, are characterized by rough feeding tolerance, strong disease resistance, good meat quality, and high fecundity (13, 14). However, studies on jejunal microbial metabolites in SB piglets remain scarce.

Metabolomics is an instant development high-throughput analysis technology that provides a more across-the-board mechanism to examine the direct relationship between metabolites and metabolic pathways by linking metabolite levels with phenotypic changes in organisms (15). Untargeted metabolomics allows for unbiased analysis and facilitates the discovery of novel biomarkers and differential metabolites, while simultaneously detecting a substantial number of metabolite signals. Li et al. analyzed the comparison of porcine jejunal tissue with porcine epidemic diarrhea virus strains and attenuated strains by proteomics (16). Zheng et al. used transcriptomics to study the effects of H2S on morphological damage and immune function in the broiler jejunum (17). However, there are few studies analyzing microbial metabolites in the jejunum using metabolomics. Therefore, we investigated jejunal microbial metabolites in LA and SB piglets using untargeted metabolomics to reveal the correlation between the immune index and immune-related differential metabolites.

## 2. Experimental materials and methods

### 2.1. Animals and sample collection

Three pregnant SB sows and three LA sows with the same test period and the third pregnancy with with 10–12 number of fetuses, respectively, were selected. They were raised in a single pen in the same environment and fed an antibiotic-free diet obtained from the National Research Council (18) (Table 1). After delivery, the sows and piglets were raised in the same column and the piglets suckled until they were 35 days old. For each sow, two piglets (six piglets per breed, twelve piglets in total) were randomly selected for slaughter. After the slaughter of 35-day-old piglets, quickly take about 2 cm of the middle segment of the jejunum, after preliminary cleaning, clean the intestinal cavity with sterile physiological saline, and collect the contents of the intestinal cavity by pushing them into the freezing tube. Then collect the jejunum sample (2–3 cm in the middle section) and transfer it to 4% polyformaldehyde solution for fixation. Twelve samples from the two pig breeds were analyzed using untargeted metabolomics. All animal procedures were approved by the Institutional Animal Care and Use Committee of Yunnan Agricultural University (No. YNAU20200022).

**Table 1:** Basic diet composition and nutrition.

**Item**	**Content**	**Nutrient level**	**Content**
Corn	75	Digestible energy (MJ/kg)	14.28
Soybean meal(grade1)	21	Crude protein	16.43
Palm oil powder	0.7	Crude fiber	2.64
Stone powder	0.3	Total calcium	0.75
Calcium hydrogen phosphate	1.5	Total phosphorus	0.66
Premix	1.5	Lysine	0.89
Total	100		

Premix is provided per kilogram of diet: Vit A 10,000 IU, Vit D 2,500 IU, Vit E 50 mg, Vit K 2.5 mg, Vit B_1_ 2.5 mg, Vit B_2_ 6 mg, Vit B_6_ 3.5 mg, Vit B_12_ 30 μg, Niacin 25 mg, D-pantothenic acid 12 mg, Folic acid 1.2 mg, Biotin 0.2 mg, Antioxidant 100 mg, Cu 150 mg, Fe 100 mg, Mn 45 mg, Zn 110 mg, Co 0.65 mg, I 0.4 mg, Se 0.3 mg.

Nutrients are all calculated values.

### 2.2. Histomorphology of the jejunum and measurement of jejunal mucosal immune indices

After sacrificing 35-day-old piglets, we quickly removed a 2 cm middle jejunum section. Hematoxylin and eosin (H&E) staining was performed according to the method described by Feldman et al. (19). The goblet cells of the jejunum were stained with Periodic Acid-Schiff (PAS) staining methods based on the method reported by Sorokin et al. (20). For determination of immune indices, we following antibodies from Servicebio Technology, Wuhan, CN: rabbit anti-ZO-1 (#GB11195, 1:200), rabbit anti-1L-1β, rabbit anti-1L-6 (#GB11113 and #GB11117, 1:800), rabbit anti-1L-10 (#GB11108, 1:300), mouse anti-TLR4 (#GB11269, 1:100), rabbit anti-TLR2 (#GB11554, 1:500), and rabbit anti-MUC-2 (#GB11344, 1:1,000) primary antibodies and an HRP-conjugated goat anti-rabbit IgG (#GB23303, 1:200) as a secondary antibody. The methods were based on those reported by Yang et al. (21).

### 2.3. Metabolite extraction

The sample (50 mg) and extraction solvent (acetonitrile-methanol-water, 2:2:1, including internal standard; 1,000 μL) were added to an Eppendorf tube, vortexed for 30 s, homogenized at 45 Hz for 4 min, and sonicated for 5 min in ice water (three times). The samples were then incubated at −20 °C for 1 h, centrifuged at 12,000 rpm at 4 °C for 15 min, and then transferred and stored in liquid chromatography-mass spectrometry (LC-MS) vials at −80 °C for ultra-high-performance LC-Q Exactive Orbitrap MS (UHPLC-QE Orbitrap/MS) analysis. Quality control (QC) samples were prepared by mixing equal aliquots of supernatants from all samples.

### 2.4. LC-MS/MS analysis

LC-MS/MS analysis was performed using a UHPLC system (1290, Agilent Technologies) and a UPLC HSS T3 column (2.1 mm × 100 mm, 1.8 μm) coupled to a Q Exactive (Orbitrap MS, Thermo). The mobile phase A was 0.1% formic acid in water for positive, and 5 mmol/L ammonium acetate in water for negative, and the mobile phase B was acetonitrile. The elution gradient was set as follows: 0 min, 1% B; 1 min, 1% B; 8 min, 99% B; 10 min, 99% B; 10.1 min, 1% B; 12 min, 1% B. The flow rate was 0.5 mL/min. The injection volume was 2 μL. The QE mass spectrometer was used for its ability to acquire MS/MS spectra on an information-dependent basis (IDA) during an LC/MS experiment. In this mode, the acquisition software (Xcalibur 4.0.27, Thermo) continuously evaluates the full scan survey MS data as it collects and triggers the acquisition of MS/MS spectra depending on preselected criteria. ESI source conditions were set as following: Sheath gas flow rate as 45 Arb, Aux gas flow rate as 15Arb, capillary temperature 320 °C, full MS resolution as 70,000, MS/MS resolution as 17,500, collision energy as 20/40/60 eV in NCE model, spray voltage as 3.8 kV (positive) or −3.1 kV (negative), respectively.

### 2.5. Data preprocessing and annotation

Raw MS data (raw) files were converted to the mzML format using ProteoWizard and processed using the R package XCMS (v3.2). The data were then filtered according to the following criteria: the number of samples containing metabolites was < 50%, all sample numbers were in one group (QC was also taken as a group). Internal standardization was performed for each sample (22). Subsequently, using default values, missing values were replaced by half of the minimum values found in the dataset (23). The preprocessing generated a data matrix consisting of retention time (RT), mass-to-charge ratio (m/z), and peak intensity values. An internal MS/MS database was used for data processing, and OSI-SMMS (v1.0, Dalian Chemical Data Solutions Information Technology Co., Ltd., China) was used for peak annotation after data processing with in-house MS/MS database.

### 2.6. Differential metabolites analysis

For multivariate analyses, including principal component analysis (PCA) and orthogonal partial least squares discriminant analysis (OPLS-DA), the OPLS variable importance in projection (VIP) score (threshold set to 1) was adopted to rank the metabolites that best distinguished the two groups. For univariate analysis, the *t*-test was used to screen for differential metabolites. Differential metabolites between the two groups were evaluated at *P* < 0.05 and VIP ≥ 1. For non-target data, when the form of adduct is considered later, the band M+H is preferred for the positive ion mode, and the band M-H is preferred for the negative ion mode.

### 2.7. Statistical analysis

Intestinal morphology and immune performance were analyzed using the Welch *t*-test in SPSS v22.0. The Wilcoxon rank test was used to calculate the diversity between groups in the R-item (v3.4.1) functional analysis. Correlations between variables were calculated using Spearman rank correlation in GraphPad Prism v7.0.

## 3. Results

### 3.1. Jejunal histomorphology

**Figure 1:**
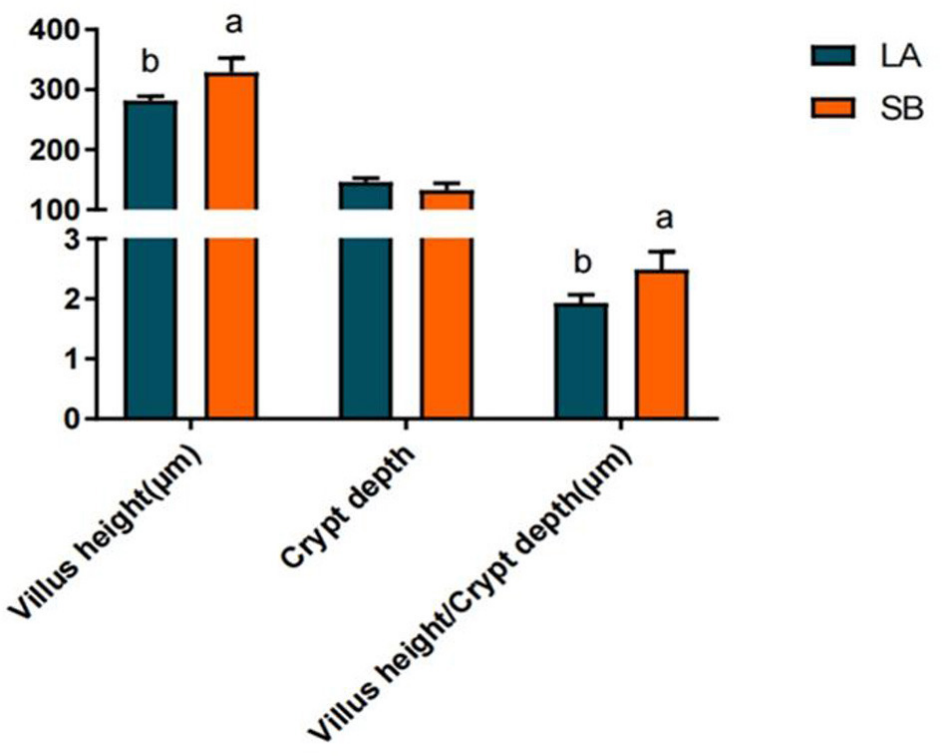
Comparison of LA and SB piglet jejunum histomorphology. (1) LA and SB respectively represent LA and SB piglets; (2) Value columns with distinctly small letters mean visible diversity (*P* < 0.05), with diverse capital letter superscripts mean remarkable diversity (*P* < 0.01). The same as below.

H&E staining results for the jejunum of LA and SB piglets are shown in Figure 1. Both jejunal villus height and villus height/crypt depth ratio were significantly higher in SB piglets than in LA piglets (*P*<*0.05*).

### 3.2. Jejunal mucosal immune factor

**Figure 2:**
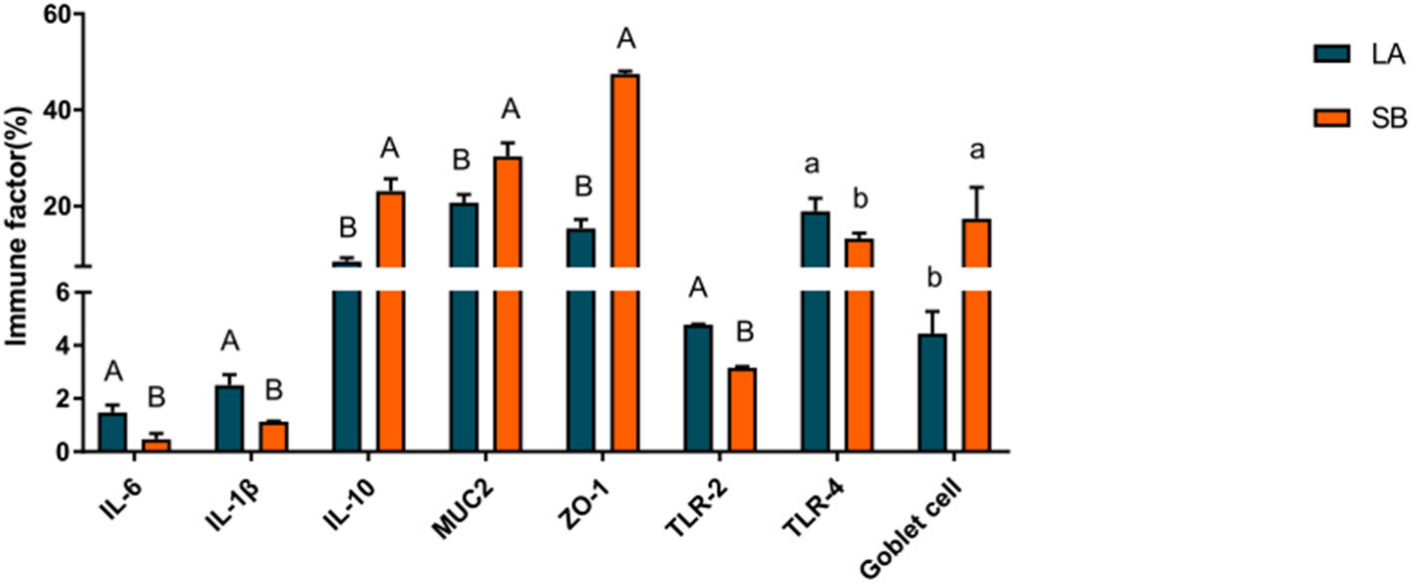
Comparison of mucosal immune factors in LA and SB piglet jejuna.

The results for jejunal mucosal immune factors of LA and SB piglets are shown in Figure 2. The results showed that the levels of IL-10, MUC2, and ZO-1 in the jejunum were markedly higher in SB piglets than in LA piglets *(P* < 0.01), while the levels of IL-6, IL-1β, and TLR-2 were markedly higher in LA piglets than in SB piglets (*P* < 0.01). Furthermore, the number of goblet cells in the jejunum was markedly higher in SB piglets than in LA piglets *(P* < 0.05).

### 3.3. Quality control of the metabolomics of jejunal chyme

The metabolomic analysis based on LC-MS/MS revealeds the difference in the metabolites of piglets' jejunal chyme in two different breeds. QC samples were used for sample quality control, as shown in Supplementary Figure S1, which depicts the overlap of BPC detected by QC sample mass spectrometry.

### 3.4. Multivariate analysis

**Figure 3:**
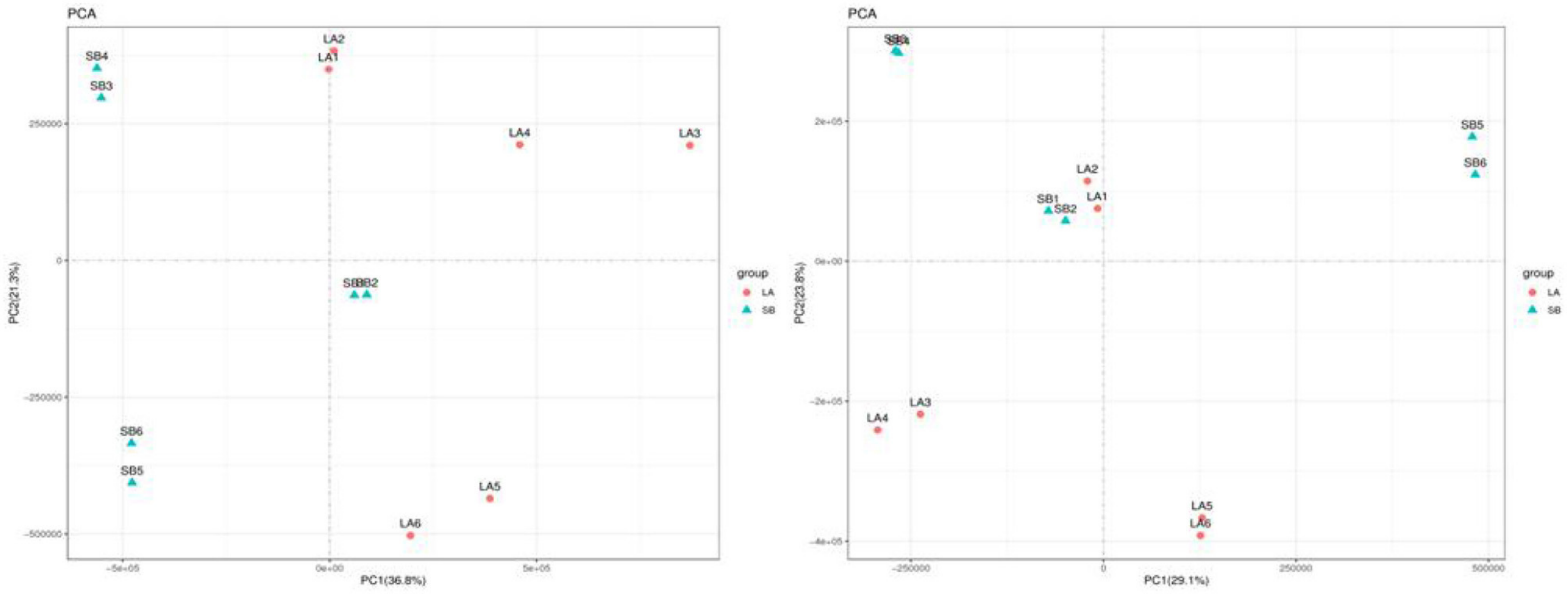
PCA map of jejunal metabolites in LA and SB piglets.

**Figure 4:**
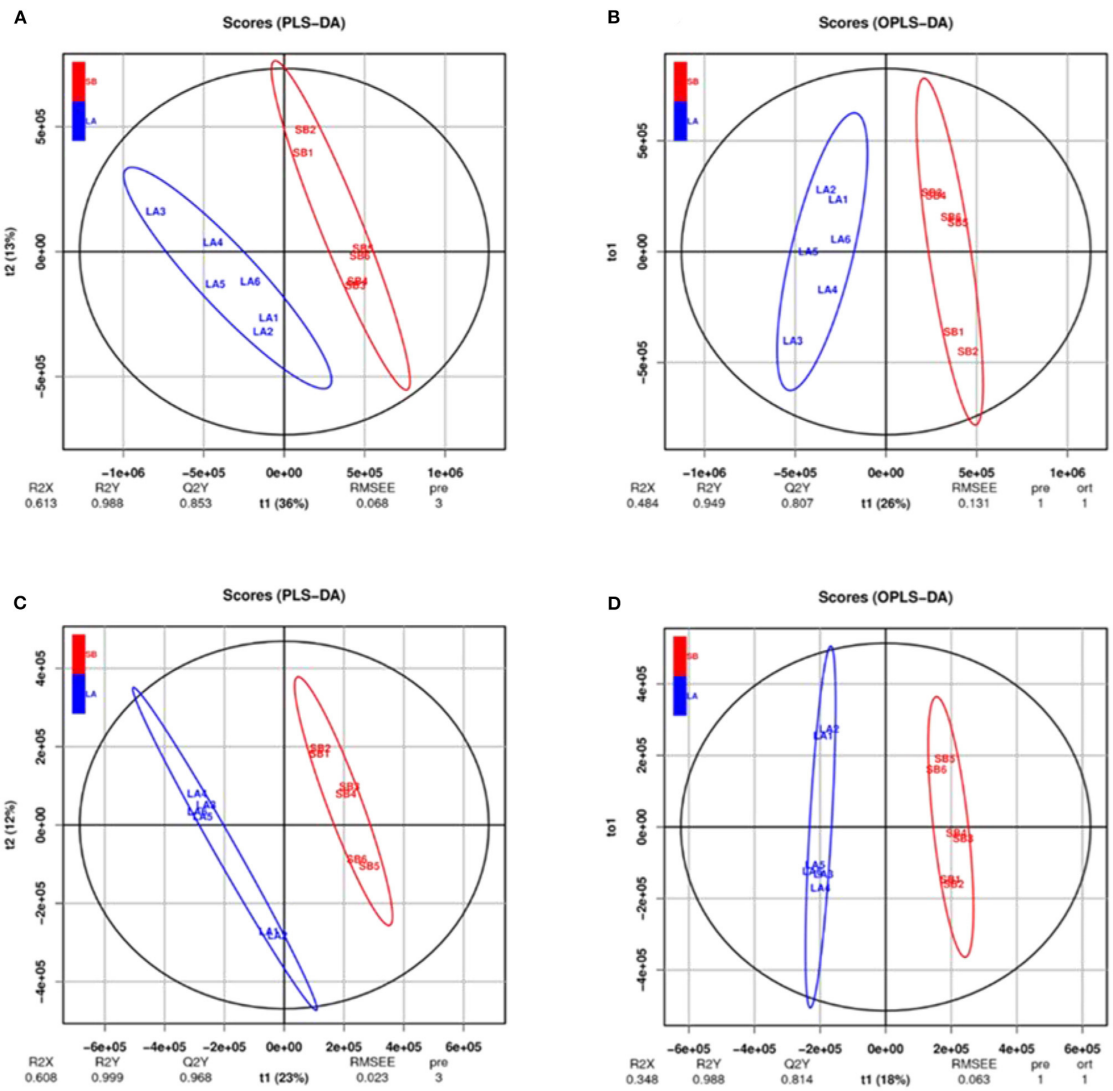
PLS-DA and OPLS-DA analysis of jejunal metabolites of LA and SB piglets under two modes. PLS-DA and OPLS-DA of relative quantification of jejunal metabolites in cationic model **(A, B)** and anionic model **(C, D)** showed no over-fitting phenomena. Thus, the model well reflects the sample characteristics for follow-up analysis.

The PCA results for the two groups are shown in Figure 3. The jejunum chyme samples from LA and SB piglets were quite different, and samples from each group (including QC) were completely separated. As shown in Figure 4 and Supplementary Figure S2, this model was reliable and could be used to screen for differential metabolites.

### 3.5. LC-MS analysis

As shown in Supplementary Table S1, 4,939 compounds were identified in each group of samples under NEG mode, including 4,655 and 284 identified by primary and secondary mass spectrometry, respectively. In total, 7,234 compounds were identified in each group of samples under POS mode, including 6,643 and 591 compounds identified by primary and secondary mass spectrometry, respectively.

### 3.6. Comparison of different jejunal metabolites between LA and SB piglets

Compared with LA piglets, the number of metabolites whose relative content was downregulated in the NEG and POS modes was much greater than that of metabolites whose relative content was upregulated in SB piglets. Compared with those in LA piglets, the relative contents of 407 and 809 metabolites were up- and downregulated in the cationic model, respectively, while the relative contents of 130 and 583 metabolites were up- and downregulated in the anionic mode, respectively (Table 2).

**Table 2:** Statistics of differential metabolites in LA and SB piglets.

**Type**	**Up-regulated**	**Down-regulated**	**All**
NEG	130	583	713
POS	407	806	1,213

### 3.7. Comparison of jejunal metabolites between LA and SB piglets

Among the top 20 metabolites identified in the NEG mode, five showed significant differences between groups, including isohyodeoxycholic acid, TDCA, chitin, alanyl-valine, and thymine (Supplementary Table S2). Cholic acid metabolites accounted for 25% of the total 20 metabolites. Isohyodeoxycholic acid levels were markedly higher in the jejunum of LA piglets than in that of SB piglets (*P* < 0.05), whereas TDCA, crustecdysone, alanyl-valine, and thymine levels were significantly lower (*P* < 0.05). Among the top 20 metabolites identified under the POS mode, four showed significant differences between groups, including bilirubin, pipecolic acid, hypoletin-8-gentiobioside, and 2-piperidone (Supplementary Table S3). Bilirubin, pipecolic acid, and hypoletin-8-gentiobioside levels were markedly higher in the jejunum of LA piglets than in that of SB piglets (*P* < 0.05), while 2-piperidone levels were substantially lower (*P* < 0.05).

### 3.8. Comparison of cholic acid metabolites between LA and SB piglets

Differences in metabolites were analyzed in the NEG mode, which showed that cholic acid metabolites accounted for 25% of the top 20 metabolites. Thus, we further compared jejunal cholic acid metabolites. As shown in Table 3, the TDCA content in SB piglets was markedly higher than that in LA piglets (*P* < 0.01).

**Table 3:** Analysis of bile acid metabolites.

**Index**	**Item**	**^a^*P*-value**	**^b^VIP**	**^c^Log2_FC**	**FDR**
NEG00147	Tauroursodeoxycholic acid	0.061	11.674	−6.129	0.269
NEG00150	Taurodeoxycholic acid	< 0.01	8.389	3.217	0.036
NEG00243	Taurochenodeoxycholate	0.097	1.004	2.749	0.330
NEG00162	Allocholic acid	0.147	0.063	16.230	0.385
NEG00111	Taurocholic acid	0.097	0.781	1.957	0.332
NEG00026	Isolithocholic acid	0.233	0.700	−0.520	0.459
NEG00045	Glycocholic acid	0.299	0.039	−0.365	0.519
NEG00032	Cholic acid	0.136	5.061	1.642	0.380
NEG00033	Chenodeoxycholic acid	0.180	0.212	3.620	0.408
NEG00036	Isohyodeoxycholic acid	0.011	22.573	−1.255	0.127
POS00350	Tauro-b-muricholic acid	0.276	0.575	1.112	0.517
POS00416	Taurochenodesoxycholic acid	0.485	0.564	−0.287	0.693
POS00169	Lithocholic acid glycine conjugate	0.335	0.114	1.254	0.572
POS00378	Chenodeoxycholic acid glycine conjugate	0.404	0.096	−1.039	0.627
POS00255	Deoxycholic acid	0.034	0.456	−8.159	0.222

^a^P-value was calculated using Student's t-test.

^b^VIP was obtained using PLS-DA model.

^c^Log2_FC is logarithm of differential expression of metabolites between two groups, with 2 as the base.

### 3.9. Correlation analysis of differential metabolites and immune factors

**Figure 5:**
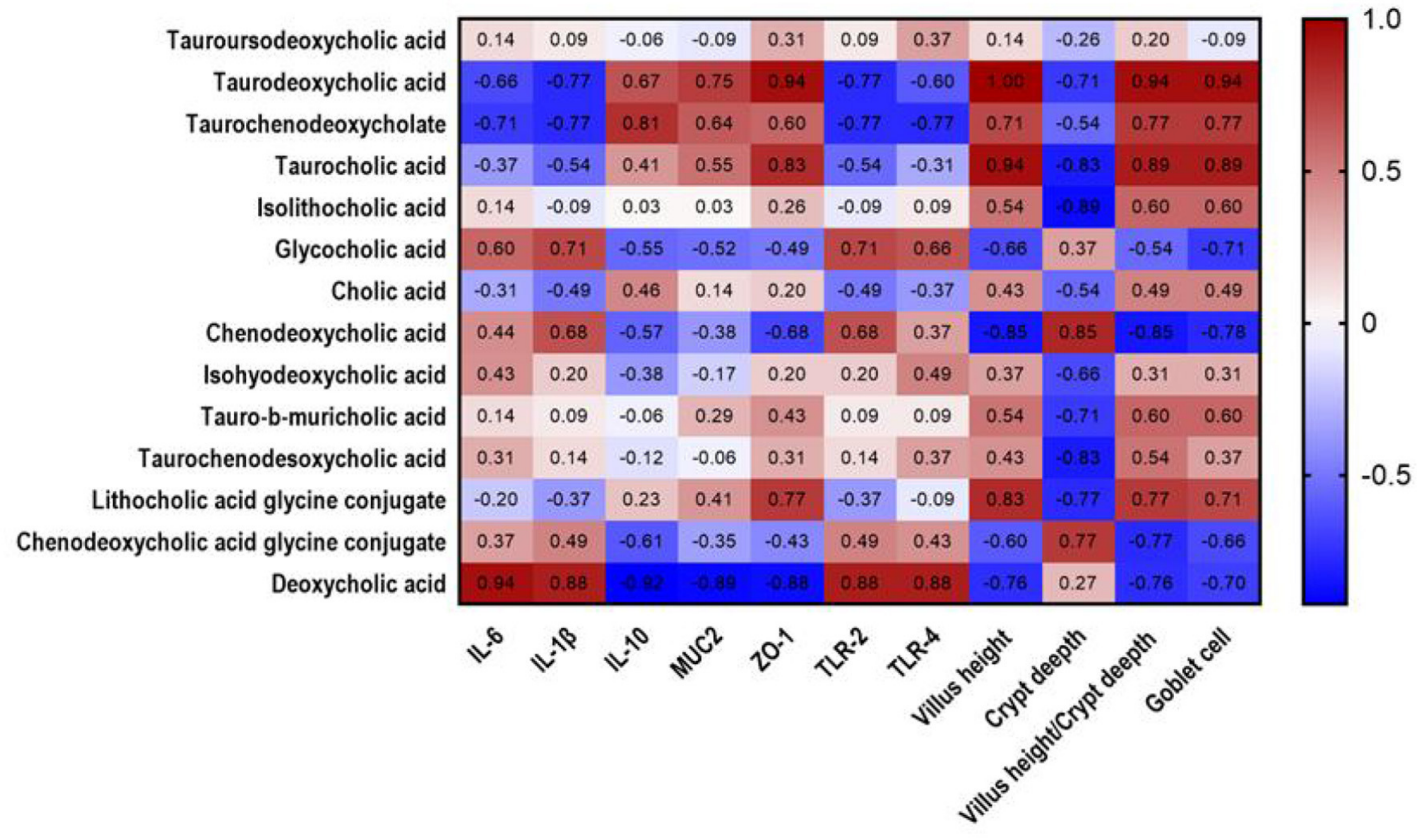
Heatmap analysis of correlation between cholic acid metabolites and mucosal immune factors in jejunal chyme of piglets. Heat plot with cholic acid metabolites in columns and mucosal immune factors in rows, showing connection between cholic acid metabolites and mucosal immune factors. Values in figure represent correlation coefficients of cholic acid metabolites and mucosal immune factors. Values >0.88 represent significant correlation. The red and blue showed the positive and negative correlation, respectively.

Spearman rank correlations among intestinal morphology, immune performance, and differential metabolites were evaluated. As shown in Figure 5, TDCA positively correlated with ZO-1 (*R* = 0.94, *P* = 0.017), villus height (*R* = 1, *P* = 0.003), villus height/crypt depth ratio (*R* = 0.94, *P* = 0.017), and goblet cell number (*R* = 0.94, *P* = 0.017).

## 4. Discussion

As a barrier between the external and strictly regulated internal environments the intestinal epithelium is essential for health (24). IL-10 plays a substantial role in maintaining mucosal homeostasis and is a key regulator of the pro-inflammatory response (25). Goblet cell secretions cover the surface of the intestinal epithelium and act as a mechanical protective barrier (26). MUC2 forms a mucus layer on the intestinal tract surface, which provides lubrication and prevents the adhesion and invasion of pathogenic bacteria (27). ZO-1 is a major component of the tightly connected functional and structural organization associated with epithelial integrity (28). Intestinal villus height and crypt depth are morphological indicators of intestinal development and maturation in piglets. A greater villus height and reduced crypt depth can positively affect absorption. The higher the ratio of villus height to crypt depth, the higher the digestion and absorption rate (29). In our study, jejunal IL-10, goblet cells, ZO-1, villus height, and villus height/crypt depth ratio were higher in SB piglets than in LA piglets, suggesting that SB piglets may have better nutrient digestion, absorption capacity, and intestinal barrier function. Len et al. reported that local pigs exhibit better digestibility of fiber diets than commercial pigs (30). Albin et al. also showed that intestinal barrier function is higher in local pigs than in Yorkshire pigs (31). Gao et al. found a higher proportion of *Lactobacillus* with anti-inflammatory functions in the jejunal microbiota of SB piglets than in that of LA piglets (32). These findings suggest that SB piglets may exhibit stronger disease resistance and that local pigs may have better digestive capacity and intestinal barrier function than commercial pigs.

Metabolites play an important role in maintaining body health, and host genetics is an important factor affecting metabolites. Based on metabolomic analysis, Lee et al. found that the metabolites in the hexane extract of *Curcuma* species differ among different varieties (33). Naka et al. also showed that blood metabolites differ between LA and Meishan pigs (34). Consistently, the OPLS-DA model scores of LA and SB piglets differed in our study. Furthermore, TDCA content was significantly lower, thus illustrating differences in jejunal chyme metabolites between LA and SB piglets.

Intestinal chyme metabolites can promote anti-inflammatory responses, participate in intestinal immune cell maintenance, and improve feed efficiency (35). Bile acids, an important group of metabolites in the intestinal chyme, account for approximately 50% of the organic content of bile (36). They are synthesized by bile alcohols in hepatocytes, are usually combined with amino acids (glycine and taurine), and are secreted into the bile and small intestine. Evidence suggests that intestinal bile acid deficiency can cause bile duct obstruction, bacterial overgrowth, and intestinal mucosal damage (37). Bile acids are material-signaling molecules that regulate various cellular and molecular functions in metabolic and non-metabolic pathways, and can produce secondary bile acids through subsequent modification by the gut microbiota or host (38, 39). Secondary bile acids include ursodeoxycholic acid, lithocholic acid, and deoxycholic acid. TDCA is a combination of deoxycholic acid and taurine found primarily in mammaliam bile (40).

Metabolomics provides great potential for phenotypic knowledge (41). Our results showed that TDCA positively correlated with ZO-1, villus height, villus height/crypt depth ratio, and goblet cell number. Previous studies have shown that TDCA exhibits potential as an ear protector after electrode insertion *via* antioxidant, anti-inflammatory, and anti-apoptotic mechanisms (42). Chang et al. reported that intravenous infusion of TDCA reduces serum proinflammatory cytokines, normalizes hypotension, prevents renal injury, and prolongs survival in mice with sepsis (43). Wang et al. reported that intestinal epithelial cells exposed to TDCA show increased cell recovery, proliferation, and anti-apoptotic protection (44). Chen et al. also showed that TDCA can prevent injury-induced intestinal apoptosis, reduce crypt cell proliferation, and maintain intestinal mucosal integrity in injured mice (45). Thus, TDCA may maintain intestinal integrity, promote intestinal digestion and absorption, and protect the intestinal tract.

## 5. Conclusion

In the present study, we found that metabolism differences between SB piglets and LA piglets, SB piglets may exhibit better jejunal immune function than LA piglets. This study provides a reference for understanding intestinal immune function in different pig breeds and for the discovery of biomarkers potentially useful for solving health issues related to pig production.

## Data availability statement

The datasets presented in this study can be found in online repositories. The names of the repository/repositories and accession number(s) can be found in the article/Supplementary material.

## Ethics statement

The animal study was reviewed and approved by the Ethics Committee of Yunnan Agricultural University (No.: YNAU20200022). Written informed consent was obtained from the owners for the participation of their animals in this study.

## Author contributions

HP designed the experiments. HP, HH, YHe, YL, YP, AL, YHu, QM, SZ, CZ, and JR performed the experiments. YHe and YL analyzed the data and wrote the manuscript. HP and HH revised this manuscript. All the authors contributed to the article and approved the submitted version.
