# Supplementary material for: Correlation analysis between jejunum metabolites and immune function in Saba and Landrace piglets
Source: Front Vet Sci. 2023 Mar 16;10:1069809. doi: 10.3389/fvets.2023.1069809 (PMC10060822; doi:10.3389/fvets.2023.1069809)
Supplement: Supplementary file 1 [file Data_Sheet_1.docx]

Supplementary Material

Supplementary Table S1 Metabolite identification results

| Type | All | MS2 | MS1 | (MS1+MS2) | Unknown |
| --- | --- | --- | --- | --- | --- |
| NEG | 29 046 | 284 | 4 655 | 4 939 | 24 107 |
| POS | 34 517 | 591 | 6 643 | 7 234 | 27 283 |

Note: All: total number of peaks obtained; NEG: negative ion mode; POS: positive ion mode. MS2: number of substances distinguished by second-order spectra; MS1: number of substances distinguished by first-order spectra; known: number of substances distinguished; unknown: number of substances undetermined.

Supplementary Table S2 Composition and content of top 20 metabolites in jejunum of different piglet breeds under NEG model

| Item | LA | SB | log2 FC | Mode |
| --- | --- | --- | --- | --- |
| Isohyodeoxycholic acid | 10.00 ± 0.07^a^ | 9.58 ± 0.11^b^ | -1.25 | - |
| (9Z)-Octadecenoic acid | 9.76 ± 0.10 | 9.58 ± 0.09 | -0.57 | - |
| Linoleate | 9.63 ± 0.13 | 9.43 ± 0.04 | -0.88 | - |
| Myristic acid | 9.50 ± 0.09 | 9.31 ± 0.05 | -0.70 | - |
| Deoxycholic acid | 9.22 ± 0.18 | 9.45 ± 0.07 | 0.51 | + |
| Tauroursodeoxycholic acid | 8.65 ± 0.49 | 7.4 ± 0.19 | -6.13 | - |
| Cholic acid | 8.62 ± 0.12 | 9.02 ± 0.17 | 1.64 | + |
| Taurodeoxycholic acid | 7.93 ± 0.06^B^ | 8.87 ± 0.1^A^ | 3.22 | + |
| D-phenyllactic acid | 8.65 ± 0.12 | 8.34 ± 0.06 | -1.23 | - |
| L-Alanine | 8.59 ± 0.03 | 8.52 ± 0.09 | -0.12 | - |
| Crustecdysone | 8.00 ± 0.13^b^ | 8.49 ± 0.09^a^ | 1.48 | + |
| Gluconic acid | 8.17 ± 0.08 | 8.02 ± 0.11 | -0.36 | - |
| Citramalate | 8.16 ± 0.04 | 7.97 ± 0.15 | -0.34 | - |
| Bilirubin | 8.14 ± 0.18 | 7.7 ± 0.06 | -1.95 | - |
| N-Acetyl-L-methionine | 8.11 ± 0.01 | 8.03 ± 0.09 | -0.12 | - |
| Alanyl-Valine | 7.54 ± 0.23^B^ | 8.19 ± 0.05^A^ | 1.42 | + |
| Uracil | 7.66 ± 0.44 | 7.06 ± 0.28 | -1.88 | - |
| Thymine | 7.46 ± 0.24^b^ | 8.11 ± 0.12^a^ | 1.57 | + |
| Valeric acid | 7.28 ± 0.16 | 7.49 ± 0.37 | 2.57 | + |
| 9-Decenoic acid | 7.84 ± 0.24 | 7.69 ± 0.12 | -0.96 | - |

Note: Figures take 10 as the logarithm of the content of base metabolites. Log2FC: logarithm of fluxionary expression of metabolites between two groups, with 2 as the base. +: up-regulated; -: down-regulated. Same below.

Supplementary Table S3 Composition and content of top 20 metabolites in jejunum of different piglet breeds under POS model

| Item | LA | SB | log2 FC | Mode |
| --- | --- | --- | --- | --- |
| Proline | 9.28 ± 0.04 | 9.26 ± 0.06 | -0.03 | - |
| 2-Methylpropanamine | 8.89 ± 0.19 | 9.05 ± 0.03 | 0.16 | + |
| Choline | 8.73 ± 0.12 | 8.75 ± 0.07 | -0.05 | - |
| Glutamate | 8.84 ± 0.04 | 8.67 ± 0.11 | -0.42 | - |
| Lysine | 8.81 ± 0.04 | 8.62 ± 0.14 | -0.36 | - |
| Amino acid (Arg-) | 8.51 ± 0.09 | 8.57 ± 0.03 | 0.09 | + |
| Betaine | 7.98 ± 0.55 | 7.54 ± 0.77 | 0.32 | + |
| Tyramine | 7.19 ± 0.67 | 8.15 ± 0.53 | 0.77 | + |
| Bilirubin | 8.52 ± 0.16^a^ | 7.90 ± 0.06^b^ | -2.43 | - |
| 1-Butylamine | 5.59 ± 0.19 | 7.06 ± 0.67 | 9.76 | + |
| Pipecolic acid | 8.50 ± 0.12^A^ | 7.96 ± 0.07^B^ | -1.90 | - |
| Beta-alanine | 8.37 ± 0.02 | 8.33 ± 0.04 | -0.10 | - |
| (+)-2,3-Dihydro-3-methyl-1H-pyrrole | 8.36 ± 0.04 | 8.18 ± 0.14 | -0.33 | - |
| D-Pipecolic acid | 8.15 ± 0.22 | 8.25 ± 0.07 | -0.09 | - |
| Pterolactam | 8.29 ± 0.04 | 8.25 ± 0.05 | -0.09 | - |
| Choline phosphate | 7.50 ± 0.59 | 7.22 ± 0.6 | -0.07 | - |
| Hypoletin 8-gentiobioside | 8.39 ± 0.07^A^ | 7.73 ± 0.05^B^ | -2.24 | - |
| 2-O-Methylcytosine | 7.76 ± 0.27 | 8.23 ± 0.07 | 0.74 | + |
| Leucine | 8.18 ± 0.04 | 7.71 ± 0.36 | -0.29 | - |
| 2-Piperidinone | 7.28 ± 0.07^B^ | 8.27 ± 0.09^A^ | 3.31 | + |

Note: Figures take 10 as the logarithm of the content of base metabolites. Log2FC: logarithm of differential expression of metabolites between two groups, with 2 as the base. +: up-regulated; -: down-regulated.


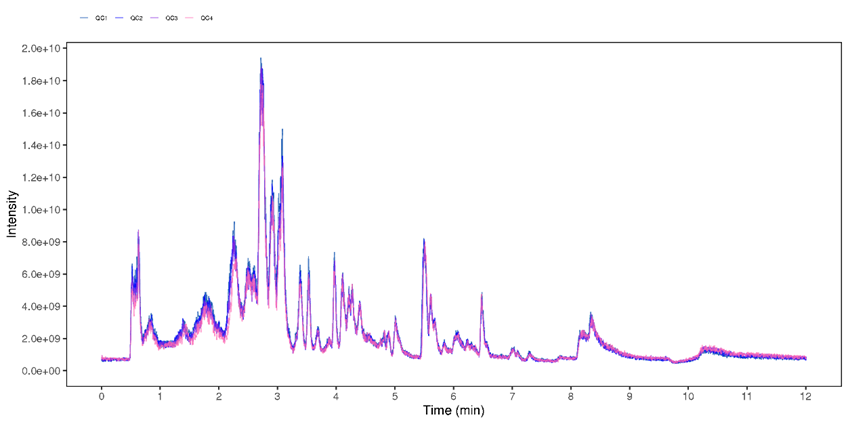


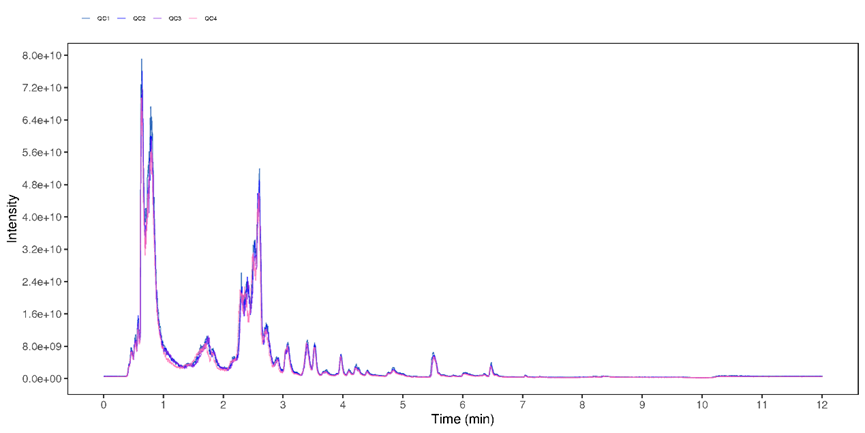


Supplementary Figure S1 BPC overlap diagram of QC sample (positive mode and negative mode) mass spectrometry


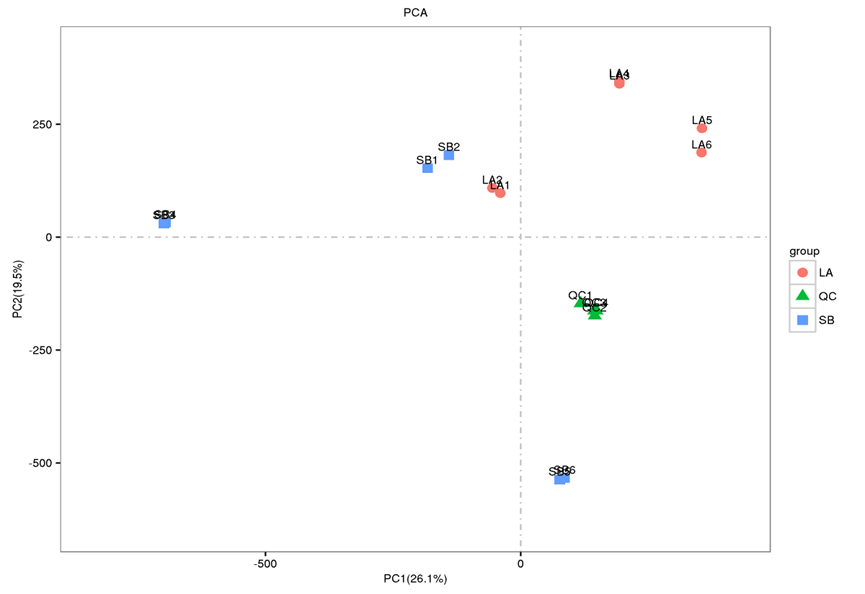


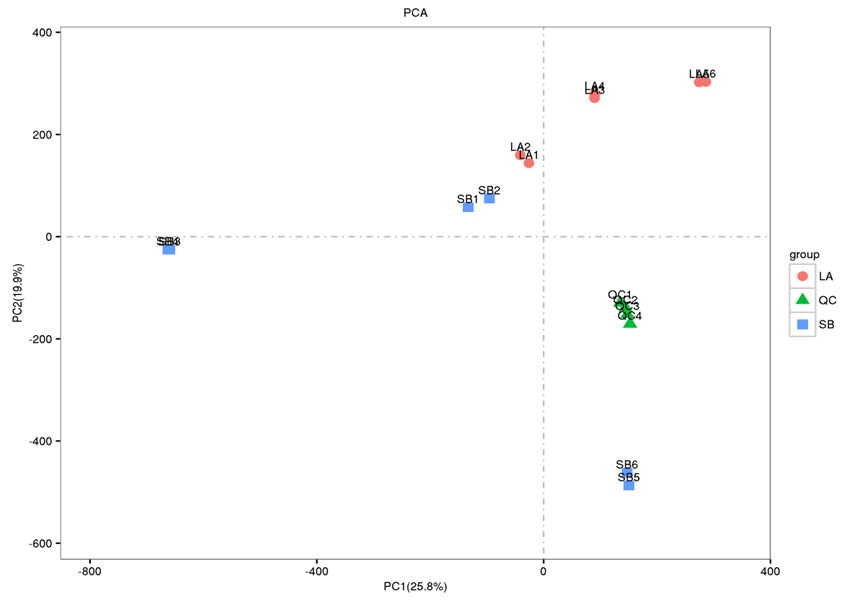


Supplementary Figure S2 principal component analysis (PCA) for all samples and quality control samples under two modes.
